# Habitat modeling of Irrawaddy dolphins (*Orcaella brevirostris*) in the Eastern Gulf of Thailand

**DOI:** 10.1002/ece3.6023

**Published:** 2020-03-04

**Authors:** Justine Jackson‐Ricketts, Chalatip Junchompoo, Ellen M. Hines, Elliott L. Hazen, Louisa S. Ponnampalam, Anoukchika Ilangakoon, Somchai Monanunsap

**Affiliations:** ^1^ Department of Ecology and Evolutionary Biology University of California Santa Cruz Santa Cruz California; ^2^ Department of Marine and Coastal Resources Marine and Coastal Resources Research and Development Center, The Eastern Gulf of Thailand Rayong Thailand; ^3^ Estuary & Ocean Science Center San Francisco State University Tiburon California; ^4^ NOAA Southwest Fisheries Science Center, Environmental Research Division/Department of Ecology and Evolutionary Biology University of California Santa Cruz Monterey California; ^5^ The MareCet Research Organization Shah Alam Malaysia; ^6^ Maharagama Sri Lanka; ^7^ Department of Marine and Coastal Resources Southern Marine and Coastal Resources Research Center Songkhla Thailand; ^8^ Present address: Independence Virginia

**Keywords:** Gulf of Thailand, habitat, Irrawaddy dolphin, *Orcaella brevirostris*, spatial management, species distribution model

## Abstract

**Aim:**

The Irrawaddy dolphin (*Orcaella brevirostris*) is an endangered cetacean found throughout Southeast Asia. The main threat to this species is human encroachment, led by entanglement in fishing gear. Information on this data‐poor species’ ecology and habitat use is needed to effectively inform spatial management.

**Location:**

We investigated the habitat of a previously unstudied group of Irrawaddy dolphins in the eastern Gulf of Thailand, between the villages of Laem Klat and Khlong Yai, in Trat Province. This location is important as government groups plan to establish a marine protected area.

**Methods:**

We carried out boat‐based visual line transect surveys with concurrent oceanographic measurements and used hurdle models to evaluate this species’ patterns of habitat use in this area.

**Results:**

Depth most strongly predicted dolphin presence, while temperature was a strong predictor of group size. The highest probability of dolphin presence occurred at around 10.0 m with an optimal depth range of 7.50 to 13.05 m. The greatest number of dolphins was predicted at 24.93°C with an optimal range between 24.93 and 25.31°C. Dolphins are most likely to occur in two primary locations, one large region in the center of the study area (11^o^54′18′′N to 11^o^59′23′′N) and a smaller region in the south (11^o^47′28′′N to 11^o^49′59′′N). Protections for this population will likely have the greatest chance of success in these two areas.

**Main Conclusions:**

The results of this work can inform management strategies within the immediate study area by highlighting areas of high habitat use that should be considered for marine spatial planning measures, such as the creation of marine protected areas. Species distribution models for this species in Thailand can also assist conservation planning in other parts of the species’ range by expanding our understanding of habitat preferences.

## Graphical abstract

We investigated the habitat of a previously unstudied group of endangered Irrawaddy dolphins (Orcaella brevirostris) in the eastern Gulf of Thailand. Depth and temperature emerged as the most important predictors of dolphin presence and group size. Using our results, we generated a map of dolphin high‐use areas that can be used to inform conservation.

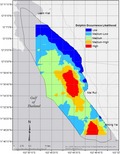

## 1 INTRODUCTION

**Figure 1:**
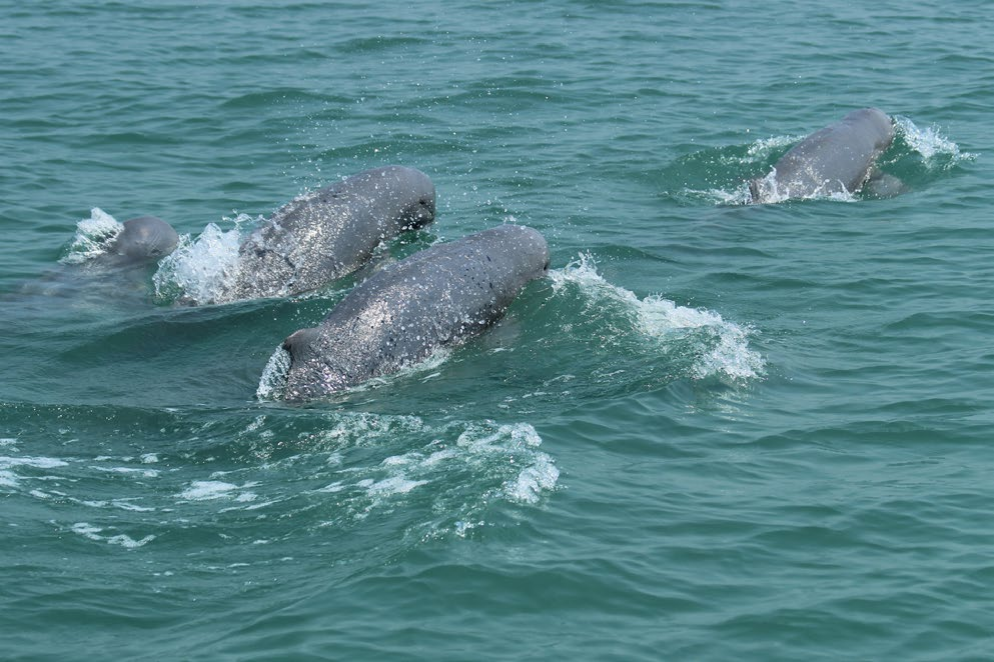
Irrawaddy dolphins (*Orcaella brevirostris*) in the Gulf of Thailand

The Irrawaddy dolphin, *Orcaella brevirostris* (Figure 1), is an endangered marine and freshwater cetacean found in South and Southeast Asia. Marine populations are patchily distributed from coastal India and Bangladesh in the northeast through Myanmar, Thailand, Malaysia, Cambodia, Vietnam, the Philippines, and Indonesia. Freshwater subpopulations inhabit the three largest Southeast Asian rivers (Ayeyarwady, Mahakam, and Mekong) and two lagoons (Chilika in India and Songkhla in Thailand). The species is classified as Endangered by the International Union for the Conservation of Nature (IUCN) due to small subpopulations, declining ranges, and increasing anthropogenic threats (Minton et al., 2017). Five recognized subpopulations (IUCN, 2013), only one of which is exclusively marine, are considered Critically Endangered. Throughout their range, this species faces numerous anthropogenic threats including gillnet entanglement, habitat degradation, pollution, noise, and boat disturbance (Minton et al., 2017). For most subpopulations, the greatest threat is entanglement in fishing gear (Beasley et al., 2007; Minton, Peter, & Tuen, 2011; Reeves et al., 2008; Smith, Beasley, & Kreb, 2003; Smith, Braulik, Strindberg, Ahmed, & Mansur, 2006). Many related and coastal dolphin species (e.g., snubfin dolphin (*Orcaella heinsohnii*), Indo‐Pacific humpback dolphin (*Sousa chinensis*)) experience similar entanglement threats (Bearzi, Fortuna, & Reeves, 2008; Bearzi et al., 2003; Karczmarski, 2000; Parra, Corkeron, & Marsh, 2006).

Marine protected areas (MPAs) that restrict fishing effort for gears with high potential impact can be a powerful tool for protecting important habitat of target species or biodiversity (e.g., Cañadas, Sagarminaga, De Stephanis, Urquiola, & Hammond, 2005; Hyrenbach, Forney, & Dayton, 2000; Kelleher, 1999). However, successful establishment of an effective MPA requires understanding the relationship between the population to be protected and its habitat as well as human uses and impacts (Cañadas et al., 2005, St. Martin & Hall‐Arber, 2008). Statistically based habitat models can provide information on preferred habitats to identify areas critical for protection and future research (Bailey & Thompson, 2009).

Little is known about Irrawaddy dolphin habitat preferences. It is one of only three cetaceans (with the finless porpoise, *Neophocaena phocaenoides*, and the tucuxi, *Sotalia fluviatilis*) able to inhabit both marine and freshwater (Smith & Jefferson, 2002). Most available data are collected from freshwater subpopulations (Baird & Beasley, 2005; Baird & Mounsouphom, 1994; Sahu, Kar, & Pattnaik, 1998; Smith & Hobbs, 2002; Smith & Jefferson, 2002; Smith et al., 2006; Smith, Shore, & Lopez, 2007; Stacey & Hvengaard, 2002; Sutaria, 2009; Pattnaik, Sutaria, Khan, & Behera, 2007; Reeves et al., 2008). In marine coastal areas, Irrawaddy dolphins are associated with warm (25°C), shallow (~6 m), and brackish to high‐salinity (>20 ppt) waters near river mouths, rarely ranging more than a few kilometers offshore (Baird & Mounsouphom, 1994; Dolar, Perrin, Gaudiano, Yaptinchay, & Tan, 2002; Minton et al., 2013, 2011; Peter, Poh, Ngeian, Tuen, & Minton, 2016; Smith et al., 2006; Smith & Hobbs, 2002; Stacey, 1996; Sutaria, 2009). This suggests that they prefer shallow nearshore areas with high nutrient input and biological productivity, likely supporting prey resources (Dolar et al., 2002; Minton et al., 2013, 2011). However, a more detailed understanding of Irrawaddy dolphin habitat characteristics is needed to establish effective conservation measures. For this purpose, we use a species distribution modeling (SDM) approach in our study of a group of Irrawaddy dolphins offshore of Trat Province, Thailand.

SDMs relate records of species occurrence to environmental predictor variables (Elith & Leathwick, 2009; Guisan & Thuiller, 2005; Redfern et al., 2006), and have been used in studies of marine mammals (e.g., Bräger, Hararaway, & Manly, 2003, Goetz, Montgomery, Ver Hoef, Hobbs, & Johnson, 2012). Properly employed and tested SDMs can play an important role in conservation by helping to illuminate species’ habitats, thus providing a framework for future research, information needed to predict species responses to environmental changes, and tools to develop effective management strategies (Bailey & Thompson, 2009; Brotons, Thuiller, Araújo, & Hirzel, 2004; Cañadas et al., 2005; Elith et al., 2006; Elith & Leathwick, 2009; Guisan & Thuiller, 2005; Redfern et al., 2006). SDMs can also predict species occurrence in difficult‐to‐access or as‐yet‐unstudied locations and species’ responses to environmental changes (Araújo, Pearson, Thuiller, & Erhard, 2005; Bailey & Thompson, 2009; Barry & Elith, 2006; Brotons et al., 2004; Elith et al., 2006; Elith & Leathwick, 2009; Guisan & Thuiller, 2005; Morin & Thuiller, 2009). Extrapolating to unstudied areas carries risk often requiring validation, however, as species–environment relationships observed in one area may not be reflected in another (Manocci, Roberts, Miller, & Halpin, 2016).

Here we examine habitat preferences of Irrawaddy dolphins in the eastern Gulf of Thailand, a subpopulation which remains unassessed by the IUCN and for which no formal habitat studies have been conducted. Local government groups are planning spatial protections for this species and need habitat and distribution information to make effective management decisions. We conducted standardized line transect surveys, collecting Irrawaddy dolphin occurrence data concurrently with data on physical and biological habitat characteristics. These data were used to develop a SDM with three goals: 1) to determine the factors influencing suitable habitat in the study area, 2) to predict dolphin distributions for use in development of conservation measures (e.g., MPA development, boating/fishing restrictions, pollution mitigation efforts, reduced dolphin entanglement risk) in the Gulf of Thailand, and 3) to provide a model for predicting Irrawaddy dolphin presence for prioritizing future sampling efforts in less studied coastal subpopulations.

## 2 METHODS

### 2.1 Field methods

**Figure 2:**
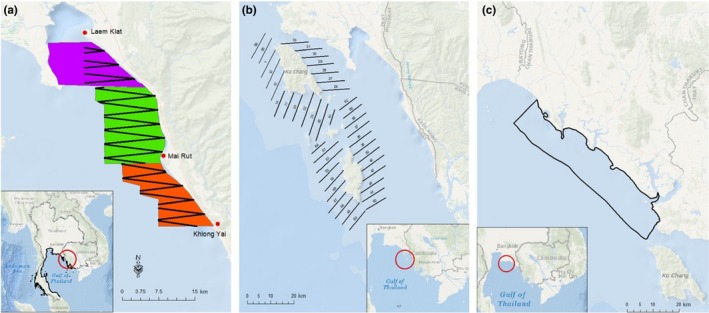
(a) Our study area off Trat Province in the eastern Gulf of Thailand including the zigzag transect lines followed for data collection with inset map showing its location in the wider Gulf of Thailand. (b) Expanded study area around the islands including parallel transect lines with inset map showing its relation to the other sites. (c) Chanthaburi study area with inset map showing its relation to the other sites

We carried out research primarily along the coast of the eastern Gulf of Thailand (Figure 2a) between the villages of Laem Klat and Khlong Yai, within Trat Province, Thailand. This subpopulation’s abundance is estimated at 423 individuals, one of the largest for this species (Hines et al., 2015). In two field seasons, 2013 and 2014, we expanded the study area to cover offshore areas surrounding three islands off the coast of Trat—Koh Chang, Koh Mak, and Koh Kut (Figure 2b)—and waters along the coast of Chanthaburi Province (Figure 2c), respectively. From 2008 to 2009 and 2012 to 2014, we carried out line transect boat surveys for three to four weeks every January and February (into March in one year) and opportunistically for one week every other month. We surveyed from a 12‐meter fishing boat, small inflatable motor boat, or 20‐meter fishing boat (Table 1). We conducted all surveys, except for the April–May 2012 fieldwork, in the dry season during the northeasterly monsoon (see Hines et al., 2015 for details). The April–May 2012 environmental data fell within the range of values collected in other years, so we included it in the full dataset rather than modeling it separately. Total area surveyed was 552 km^2^ in Trat Province, 2,127 km^2^ around the islands, and 815 km^2^ in Chanthaburi.

**Table 1:** Dates, location, and duration of data collection as well as equipment used

Year	Area	Month(s)	Survey days	Depth	Temperature	Salinity	Turbidity	Chl *a*	pH
2008	Trat	February–March	18	Davis Instruments Portable Water Depth Sounder Gauge	YSI Model 30 Handheld Salinity, Conductivity, and Temperature System		Secchi disk	na	na
2009	Trat	January	17				LaMotte Model 2020 Turbidimeter		
2012	Trat	January	20						
	Trat	April–May	4						
2013	Islands	January	11		Eureka Environmental Manta 2 Water Quality Multiprobe				
	Trat	January–February	13						
2014	Chanthaburi	January	5	HawkEye Handheld Sonar System					Manta 2
	Trat	January	13						

Environmental data were collected at time of sighting or at every 1‐km^2^ grid cell and included sea surface temperature, depth, salinity, turbidity, pH, and chlorophyll *a* (Table 1). All of these variables have been shown to inform marine mammal distribution models (Redfern et al., 2006; Torres, Read, & Halpin, 2008). These factors can limit dolphin distribution due to potential physiological constraints (e.g., temperature, salinity, and pH limits), prey availability (e.g., depth, chlorophyll *a*), and the influence of turbidity on visual capture ability and water quality. We included a binary variable indicating calf presence during a sighting as an independent variable to test whether calf presence influenced dolphin group size or presence.

### 2.2 Analytical methods

**Figure 3:**
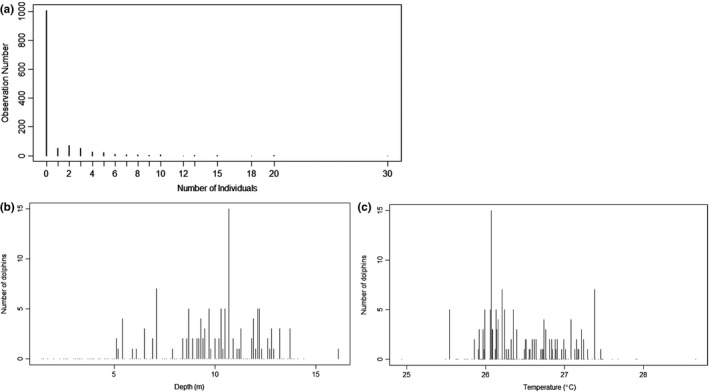
(a) Sightings frequency plot, showing the data to be highly zero‐inflated, (b) histogram of dolphin sightings versus depth, (c) histogram of dolphin sightings versus temperature. Panels b) and c) are from the dataset used for the best model framework, showing that sightings appear concentrated at medium depths and temperatures

We first measured distances to the coastline and river mouths for each environmental data point using ArcGIS (ESRI, 2014). We carried out all subsequent analyses in R version 2.13.1 (R Development Core Team, 2009–2013). We identified and removed outliers (points more than three standard deviations from the mean, according to the *Z*‐value test) in each data category (e.g., sightings, depth, and salinity) (Aggarwal, 2013; Hodge & Austin, 2004). We binned turbidity and chlorophyll *a* into high, medium, and low categories using a Jenks natural breaks classification because variability was high between years, low within years, and non‐normally distributed. We used a pair plot to initially explore the data and identify linear relationships. Next, we ran both Moran’s I and Mantel tests to determine if sightings were spatially autocorrelated (Dray, Dufour, & Thioulouse, 2016; Paradis et al., 2015). Neither analysis found significant clustering of sightings (*p* > 0.05). We tested variables for collinearity using variance inflation factors (VIF) with a cutoff value of 3 (Naimi, 2015; Zuur, Ieno, Walker, Saveliev, & Smith, 2009), resulting in removal of the variable “distance to coastline.” Frequency plots showed sightings data were highly zero‐inflated and overdispersed (Figure 3a; mean = 0.78, variance = 4.91). We chose a hurdle model, which models data in two components. The zero component models data as binary with a binomial distribution (zeros vs. all nonzero counts) and the truncated count component models just nonzero counts using a Poisson, negative binomial, or geometric distribution (Hu, Pavlicova, & Nunes, 2011; Zeileis, Kleiber, & Jackman, 2008; Zuur et al., 2009). Hurdle models are an appropriate choice for this study given the nature of our data and their past use in modeling the distribution of marine mammals (e.g., Goetz et al., 2012; Gowan & Ortega‐Ortiz, 2014; Ver Hoef & Jansen, 2007). The frequency curve for the sightings data (Figure 3a) closely resembles a negative binomial distribution with mean = 1 and dispersion parameter (*k*) = 0.1 (Zuur et al., 2009). Therefore, we analyzed our count data using a negative binomial distribution. This model family assumes that separate ecological processes influence presence/absence and number of individuals where the species is present (Zuur et al., 2009).

Hurdle models do not handle missing data well and *k*‐fold cross validation failed on a model including all years and all variables. Due to uneven data collection across years and missing values caused by instrument error, data availability was uneven across years (Table 2). Therefore, we separated the data into five smaller datasets (Table 3). Four datasets included all data points for each year and thus left out variables with missing values. The fifth dataset held only points for which all variables were measured and was smaller than the prior four. Because there were no sightings around the islands or Chanthaburi, we left these data out of the analysis. We explore differences and potential reasons for this lack of sightings in the Discussion.

**Table 2:** Data availability in the Trat study area

Data	Total entries	Depth	Temperature	Salinity	Turbidity	Chl *a*	pH	Dist. to river mouth
February–March 2008	279	279	279	279	121	0	0	279
January 2009	218	218	218	218	218	0	0	218
January 2012	203	203	203	203	203	0	0	203
April–May 2012	35	35	35	35	35	0	0	35
January–February 2013	174	174	174	174	174	174	0	174
January–February 2014	185	185	185	185	185	185	185	185
TOTAL	1094	1094	1094	1094	936	359	185	1094

**Table 3:** Model frameworks organized in order to maximize data used in the models. Framework 5 is reduced such that it only contains entries with all variables

Model framework	Variables	Data
1	Depth	2008
	Temperature	2009
	Salinity	January 2012
	Distance to river mouth	April–May 2012
	Calves	January–February 2013
	Year	January–February 2014
2	Depth	2009
	Temperature	January 2012
	Salinity	April–May 2012
	Turbidity	January–February 2013
	Distance to river mouth	January–February 2014
	Calves	
	Year	
3	Depth	January–February 2013
	Temperature	January–February 2014
	Salinity	
	Turbidity	
	Chlorophyll *a*	
	Distance to river mouth	
	Calves	
4	Depth	January–February 2014
	Temperature	
	Salinity	
	Turbidity	
	Chlorophyll *a*	
	pH	
	Distance to river mouth	
	Calves	
5	Depth	2008
	Temperature	2009
	Salinity	January 2012
	Turbidity	April–May 2012
	Distance to river mouth	January–February 2013
	Calves	January–February 2014
	Year	

We fit the data to a suite of hurdle models (Jackman, Tahk, Zeileis, Maimone, & Fearon, 2015), first using all variables within each framework, then dropping terms sequentially and performing model selection tests and evaluations (detailed below) to determine which configuration of terms resulted in the best model. We used Akaike’s information criterion (AIC) and *k*‐fold cross validation (with 10 folds) (Alfons, 2012) to choose the best model within each framework (Johnson & Omland, 2004; Kadane & Lazar, 2003; Kohavi, 1995; Redfern et al., 2006; Refaeilzadeh, Tang, & Liu, 2009; Zuur, Ieno, & Smith, 2007). We supplemented these criteria with a likelihood ratio test (Hothorn et al., 2015) to compare each reduced model to the full model within each framework (nested models), with a threshold of *p* > 0.05 (Johnson & Omland, 2004).

After choosing models from each framework, we evaluated each model component (presence/absence and count) using the area under the curve (AUC) of the receiver operating characteristic (ROC) plot (Franklin, 2009, Sing, Sander, Beerenwinkel, & Lengauer, 2015). We also calculated McFadden’s pseudo‐*R* ^2^ (*ρ* ^2^), a goodness‐of‐fit measure, as a method of model evaluation (McFadden, 1978). From these assessments, we chose a final set of models. To choose the final model, we ran all parameters as linear, except for depth and temperature, which were modeled using a quadratic (Figure 3b,c). After choosing the final model, we used the “predict” function in the PSCL package (version 1.4.9; Jackman et al., 2015) to obtain predicted probability, count, and overall fitted values from the model. We used these values to create an interpolated surface using an ordinary krige with a 3 × 3 smoother in ArcGIS (ESRI, 2014). We further determined optimal values of the significant variables identified by the models: We first set all environmental variables in the final model to their average values, then varied only the significant variables, using the “predict” function to make predictions for the relevant model part (zero or count). These optimal values were used to describe the preferred habitat of this species as well as to compare our results with temperature and depth preferences obtained in other parts of its range. To determine the areas most highly used by Irrawaddy dolphins along the Trat Province coast, we classified overall fitted values by Jenks natural breaks into five classes of dolphin occurrence likelihood (high probability of presence and large group size) in ArcGIS (ESRI, 2014).

## 3 RESULTS

**Figure 4:**
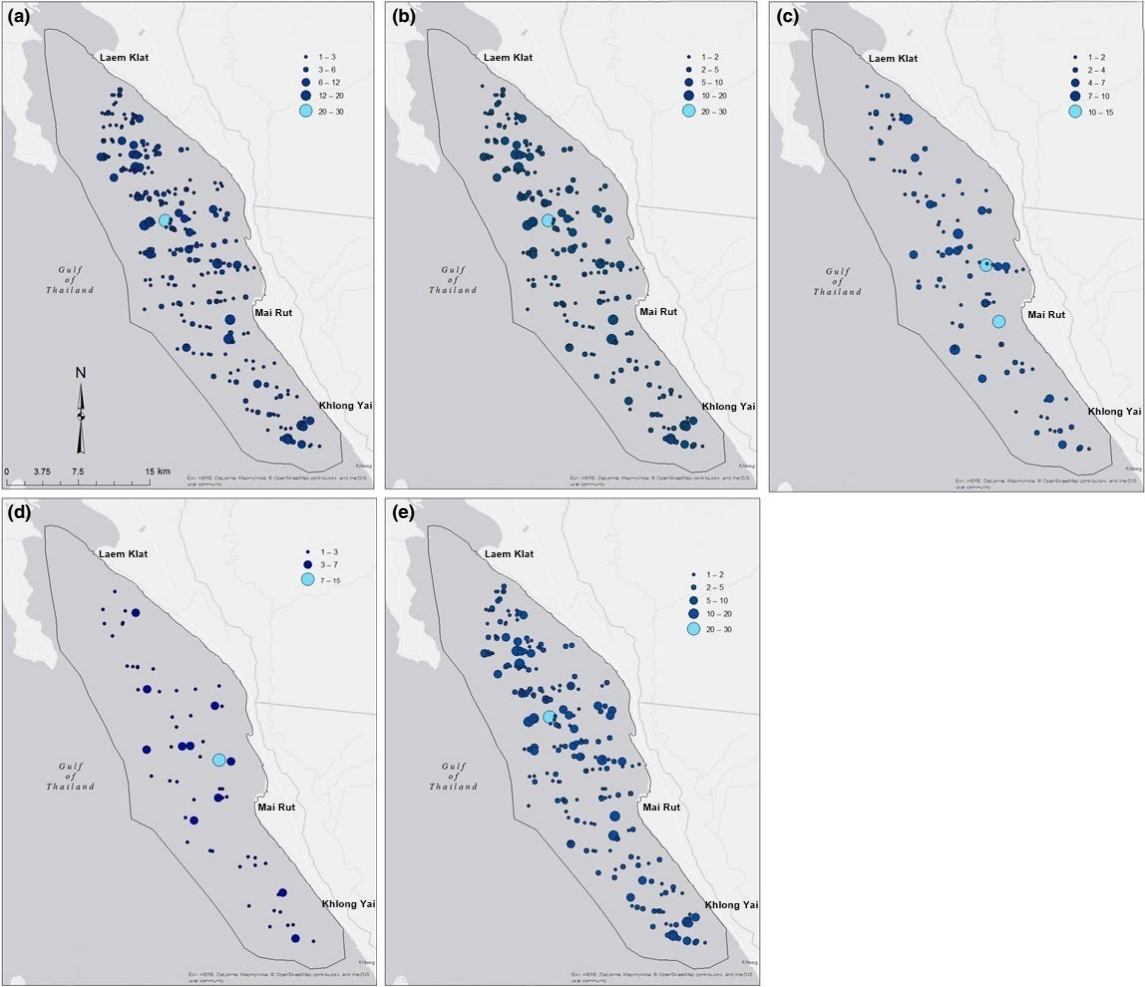
Sightings data used for the five hurdle models. (a) Model 1 data, which include sightings from between 2008 and 2014. (b) Model 2 data, which include sightings from between 2009 and 2014. (c) Model 3 data, which include sightings from between 2013 and 2014. (d) Model 4 data, which include sightings from 2014. (e) Model 5 data, which include sightings from between 2008 and 2014, only containing entries which included all variables. Largest groups are a lighter blue and were all observed off the coast near the town of Mai Rut

Dolphins were encountered in every part of the study area except around the islands or off the coast of Chanthaburi. The environment was similar in these regions, with the notable exceptions of depth and distance to river mouth (Table 4). Most observed groups were small (1–5 individuals), but a few large groups were observed in Laem Klat and Khlong Yai, and the largest were observed in Mai Rut (5–15 and 15–30 individuals, respectively; Figure 4). Average group size was 3.77 individuals (*SD* = 3.55, range = 1–30).

**Table 4:** Mean of environmental variables in each study area

	Trat	Islands	Chanthaburi
Average temperature (^°^C)	28.73	29.3	27.83
Average chlorophyll *a*	1.62	1.74	1.48
Average salinity (ppt)	31.2	29.93	32.88
Average depth (m)	8.22	16.42	9.68
Average turbidity	1.64	2	1.95
Average pH	7.89	na	8.14
Average distance to river mouth (km)	7.58	26.62	10.58

Our final chosen model was framework 4 with the quadratic depth term, containing data from 2014 only and including salinity, turbidity, calves, a quadratic depth term, and distance to river mouth in the zero component and temperature, turbidity, chlorophyll *a*, and pH in the count component (Table S3).

**Figure 5:**
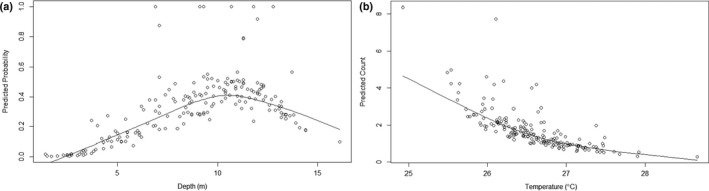
Scatterplots with lowess lines showing (a) a positive relationship between predicted probability of dolphin occurrence and depth until around 11 meters, at which point the relationship becomes negative, and (b) a negative relationship between predicted dolphin number and temperature

Both the first (linear)‐ and second (nonlinear)‐order depth terms were significant predictors for the zero component (*p* < 0.005), while temperature was a significant predictor for the count component (*p* < 0.005) (Table S3). Predictions from the zero component (probabilities of dolphin presence) show a positive relationship with depth at 10.5 meters and below, with a negative relationship above 10.5 m (Figure 5a). Predictions from the count component (dolphin numbers) show a steady negative relationship with temperature, with multiple dolphins predicted at 25°C and almost none at around 28.6°C, the highest temperature recorded (Figure 5b).

**Figure 6:**
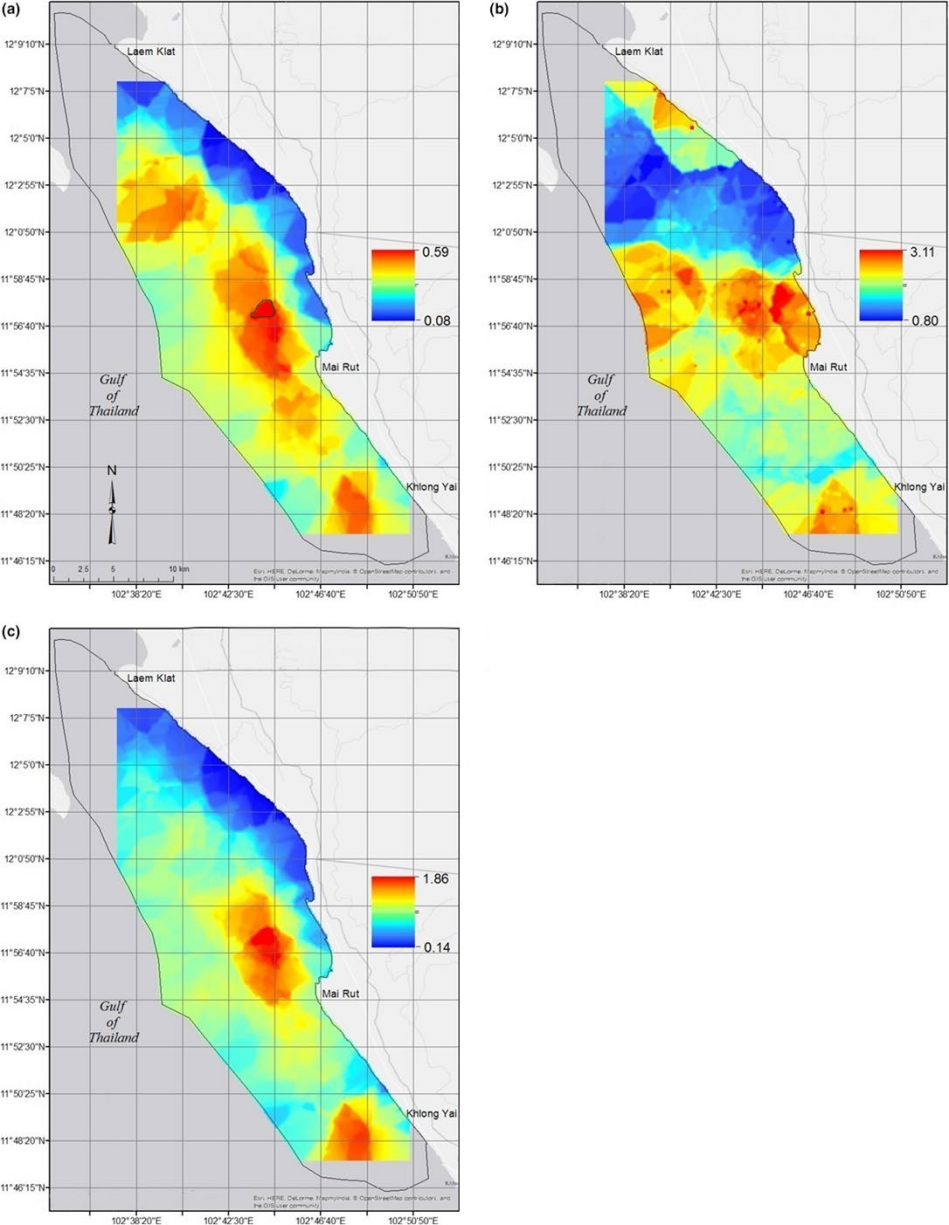
(a) Predicted probability of dolphin occurrence, indicating three distinct areas of high probability, (b) predicted dolphin counts, showing one major area of dolphin congregation and two minor areas, one of which is likely to support large groups, given the probability results shown in a, and (c) fitted model predictions, clearly showing two distinct areas of high probability of dolphin presence and large group size (together dolphin occurrence likelihood). We employed kriging and a 3 × 3 smoother to the data, so the ranges are smaller than those predicted by the model (0.004–1 for probability, 0.29–8.36 for counts, and 0.01–4.16 for fitted predictions)

**Figure 7:**
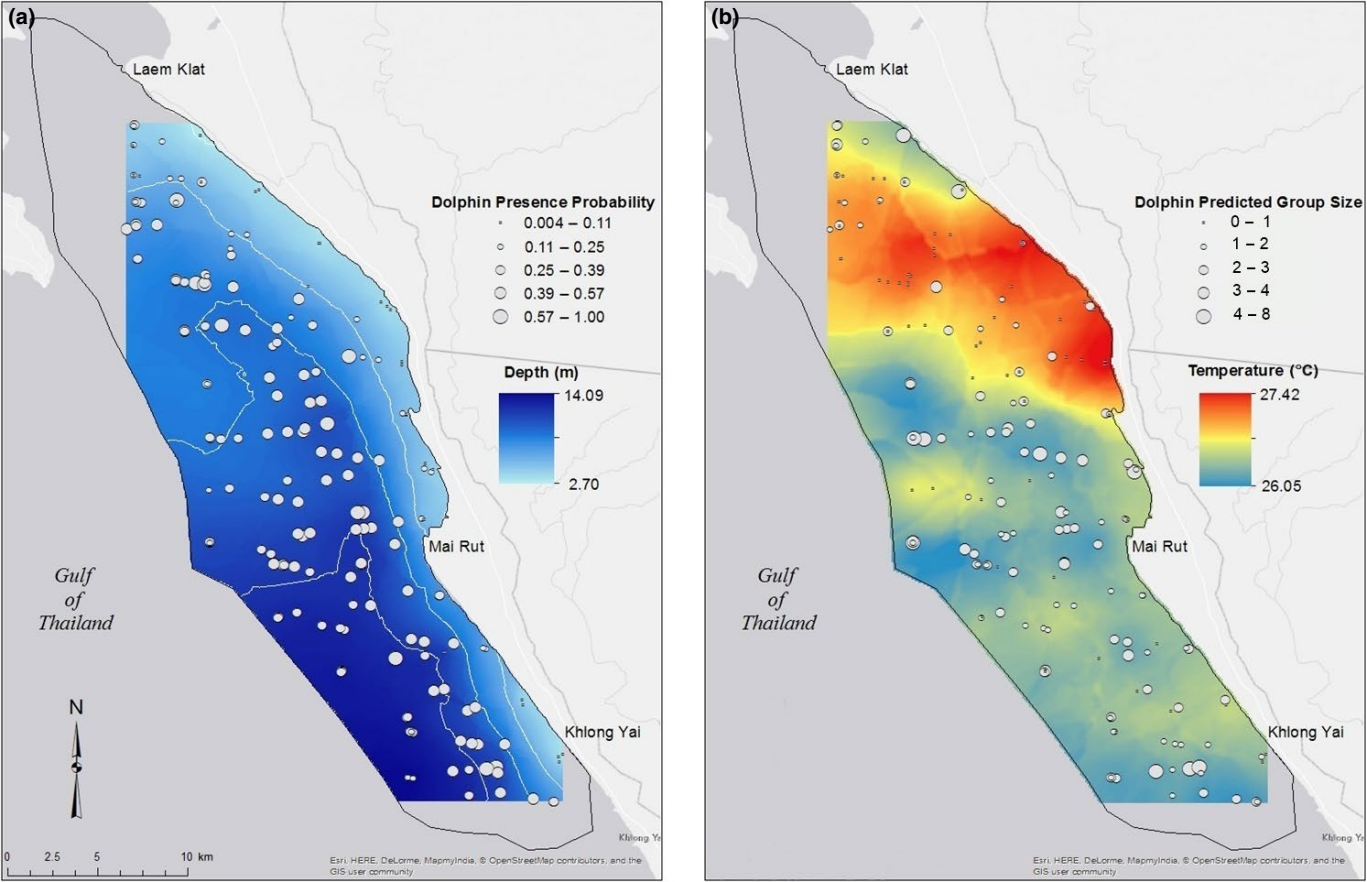
Maps of (a) depth in the study area with point values of presence probability, showing that areas of high predicted dolphin occurrence probability coincide with areas of medium depth (1.4–16.1 m), and (b) temperature in the study area with point values of predicted group size, indicating that higher numbers of dolphins are most likely found in lower temperature areas. I applied kriging and a 3 × 3 smoother to the raw data in order to obtain a smooth surface. These calculations resulted in ranges of depths and temperatures that were smaller than the recorded ranges (1.4–16.1 m and 24.93–28.66°C, respectively)

Dry‐season dolphin presence probability was greatest just off the coast from approximate latitudes 11^o^56′57′′N to 11^o^57′48′′N, between approximately 3.0 and 6.0 kilometers offshore (circled in green in Figure 6a). This small patch of high probability is surrounded by a larger patch of slightly lower probability extending to around 7.5 kilometers offshore between approximate latitudes 11^o^54′21′′N and 11^o^57′48′′N. Two other lower probability patches lie between 2.5 and 6.5 kilometers offshore from approximate latitudes 11^o^47′31′′N to 11^o^50′5′′N and between 5 and 12.5 kilometers offshore from approximate latitudes 12^o^0′30′′N to 12^o^3′28′′N, where water remains shallower farther offshore. The farthest offshore we encountered dolphins was 11.04 kilometers in the central section of the study area (11^o^55′12′′N, 102^o^40′12′′E). They are unlikely to be found in the shallowest nearshore waters between approximate latitudes 11^o^56′43′′N and 12^o^7′32′′N. These results are supported by a map of depth in the study area, showing that the areas of highest presence probability are in locations with mid‐range depths (~5‐10 m) (Figure 7a).

The largest dolphin group sizes were predicted in the central part of the study area, between approximately 1.0 and 5.0 kilometers offshore and approximate latitudes 11^o^55′28′′N and 11^o^59′10′′N, with a smaller patch at around 9.0 kilometers offshore between approximate latitudes 11^o^57′32′′N and 11^o^59′31′′N (Figure 6b). Two other areas with slightly lower predicted group sizes occur between approximate latitudes 11^o^47′29′′N and 11 ^o^49′60′′N, around 3.0 to 7.0 kilometers offshore and nearshore from approximate latitudes 12^o^5′12′′N to 12^o^7′14′′N. However, given the low probability of dolphin occurrence in nearshore northern waters, this third area is unlikely to support large groups of dolphins. A map of temperature (the strongest predictor of dolphin group size) in the study area strongly supports model predictions (Figure 7b).

Optimal depths (dolphin presence probability > 0.50) are between 7.50 and 13.05 meters, with the highest probability of dolphin occurrence (0.6395) at around 10.0 meters. Optimal temperature range (>5 dolphins predicted) is 24.93–25.31°C, with the highest number of dolphins (>7) predicted at 24.93°C. Above 26.99°C, <1 dolphin is predicted.

Overall fitted values suggest two small areas of both high probability of dolphin presence and large group size (>5 individuals) (Figure 6c). These are between approximately 3.0 and 6.0 kilometers offshore from approximate latitudes 11^o^56′6′′N to 11^o^57′56′′N and 2.5 and 7.5 kilometers offshore between approximate latitudes 11^o^47′27′′N and 11^o^49′19′′N.

**Figure 8:**
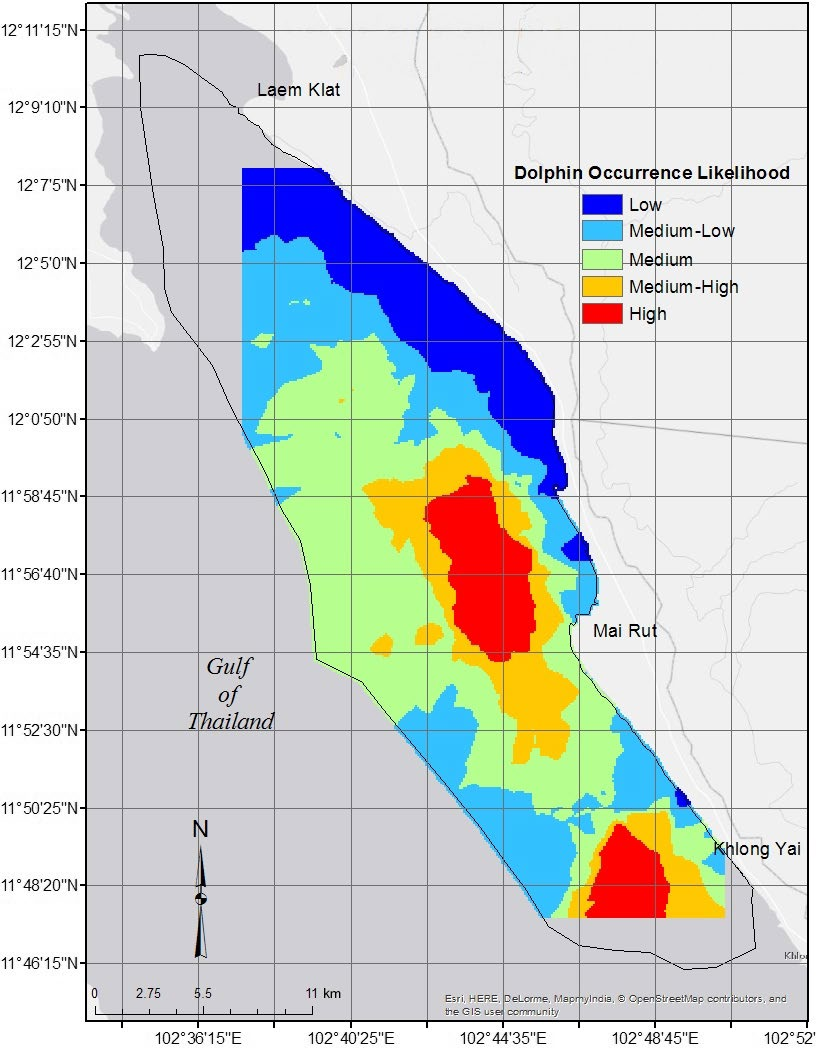
Map of dolphin occurrence likelihood. Red indicates areas of highest dolphin occurrence likelihood that, if protected, would preserve the greatest number of dolphins. Orange indicates potential buffer zones surrounding the high likelihood areas

Classification resulted in two areas of high dolphin occurrence likelihood. These surrounded the areas in Figure 6c, stretching from 11^o^54′18′′N to 11^o^59′23′′N in the middle of the study area, approximately 1.5 to 7.0 km offshore of Mai Rut, spanning longitudes 102^o^45′24′′E to 102^o^42′18′′E and from 11^o^47′28′′N to 11^o^49′59′′N offshore of Khlong Yai from 102^o^46′51′′E to 102^o^49′4′′E (Figure 8).

## 4 DISCUSSION

### 4.1 Dolphin–habitat relationships in the Gulf of Thailand

In the eastern Gulf of Thailand, dry‐season dolphin presence was most strongly predicted by depth, while temperature strongly predicted group size. This indicates preference for relatively cool waters of intermediate depth. Geographically, this places Irrawaddy dolphins at 1.5 to 7.5 km from shore (Figure 6). Protected area planning with protective zoning based on these areas would likely have the greatest chance of protecting this population (Batisse, 1982; Day et al., 2012; Hooker, Whitehead, & Gowans, 1999; Hyrenbach et al., 2000; Kelleher, 1999; Lausche, 2011). Buffer zones could be formed surrounding the two areas, between approximate latitudes 11^o^51′34′′N and 12^o^0′10′′N, around 0.25 to 10.5 kilometers offshore of Mai Rut, and from approximate latitudes 11^o^47′28′′N to 11^o^50′22′′N, approximately 1.5 to 10.5 kilometers offshore of Khlong Yai (Figure 8) to protect animals traveling to and from core areas (Batisse, 1982; Day et al., 2012; Hooker et al., 1999; Hyrenbach et al., 2000; Kelleher, 1999; Lausche, 2011). Bycatch of cetaceans is high in the eastern Gulf of Thailand, with 12% of fishers interviewed reporting knowledge of cetacean bycatch. While willingness to change fishing gear is low (Teh, Teh, Hines, Junchompoo, & Lewison, 2015), Thai fishing communities consider marine conservation an important goal and are willing to work toward bycatch reduction (Teh et al., 2015). The Thai government is currently working to balance protection of dolphins with the needs of small‐scale fishers. The Thai Department of Marine and Coastal Resources is currently working on creating the first MPA along the Trat Province coast, with an emphasis on conservation of Irrawaddy dolphins. Placing a zonal MPA (divided into zones of regulated use) that maximizes protection in the region where dolphins are most abundant could lead to significant bycatch reduction, although effective spatial protections for highly mobile species such as cetaceans can be difficult (Embling et al., 2009; Habtemariam & Fang, 2016).

### 4.2 Comparison with other locations

Prior studies on Irrawaddy dolphin distribution and habitat have been informal, based upon average environmental variables at dolphin sighting locations, with the exception of work conducted in Kuching Bay, Sarawak, Malaysia (Minton et al., 2013; Peter et al., 2016). Marine Irrawaddy dolphin habitat has been assessed in bay, delta, estuary, and coastal areas of Bangladesh, Cambodia, Indonesia, Malaysia, Myanmar, the Philippines, and now Thailand (Table S4). Comparisons between our site and other locations can help managers make inferences about potential reasons for absence in some areas while building a more complete picture of Irrawaddy dolphin habitat. While this study confirms the general trends detected in other study areas, there are a few aspects in which our results differ from those of other studies. In coastal areas of Cambodia, Indonesia, Malaysia, and the Philippines, dolphins were encountered within a few kilometers of the coast and river mouths, in relatively wide depth ranges (13.32m average spread, 21m maximum), narrow temperature ranges (average range 5°C), narrow turbidity ranges, moderate salinity ranges (average range 17.28 ppt), and somewhat basic pH (Table S4) (Beasley & Davidson, 2007; Dolar et al., 2002; Kreb & Budiono, 2005; Minton et al., 2011; Ponnampalam, 2012, 2013; Ponnampalam, Kuit, & Chong, 2014; Smith et al., 2004; Yanuar et al., 2011). Our depth and temperature ranges were narrower than those found in other studies (5.55 m and 2.06°C, respectively). This may be due to more uniform conditions, narrower dolphin preference in this region, or a combination of factors that our model was unable to address.

In bays of East Kalimantan, Indonesia, and Sarawak, Malaysia, a combination of anecdotal sightings reports and statistical methods (Kruskal–Wallis U tests, Fisher’s exact test), indicated that dolphins prefer nearshore, somewhat brackish and turbid waters of widely varying depths (Table S4) (Kreb & Budiono, 2005; Kreb & Rahadi, 2004; Minton et al., 2013; Peter et al., 2016). Average depth of dolphin sightings was deeper than our predicted optimal depth (Kreb & Budiono, 2005; Kreb & Rahadi, 2004), suggesting that a different variable, likely salinity (Minton et al., 2013; Peter et al., 2016), is a stronger driver in this habitat than in the Gulf of Thailand (Table S4).

In the outer Sundarbans Delta of Bangladesh and deltas of East Kalimantan, Indonesia, dolphin observations occurred in narrow depth (average spread 9.1 m) and temperature ranges (3.6°C), but wide turbidity and salinity ranges (Table S4) (Kreb & Budiono, 2005, Smith et al., 2005). Average depths were shallower than identified for the Gulf of Thailand (average 6.23m). Temperatures at which dolphins were found were generally lower than those optimal in the Gulf of Thailand (average 23.7°C). This may be due to habitat availability (e.g., no temperatures below 24.93°C were recorded in any region of our study area) or the interaction of factors that were not discernible to our model.

### 4.3 Potential reasons for absence in the islands and Chanthaburi

We did not see dolphins in the islands or Chanthaburi, but we can compare environmental measures between these areas and Trat. As Table 4 shows, average values of environmental variables are mostly similar. For temperature (Table 5), chlorophyll *a*, and salinity, Trat values fall between the values of the other two areas. The Trat study area is, however, characterized by much lower depths (Table 5), turbidity, and distances to river mouth than the other areas (Table 4) and is much less developed than Chanthaburi, with lower levels of fishing activity and industrial development. River mouths were less prevalent in the islands and Chanthaburi, resulting in sampling locations much farther from river mouths in those areas (average: 26.62 km and 10.58 km, respectively) than in Trat (average: 7.58 km) (Table 6). In Trat, sightings did not occur more than 14.17 kilometers from a river mouth. The potential importance of proximity to river mouths is supported by the fact that distance to river mouth is considered a reliable indicator for this species in other systems (Baird & Mounsouphom, 1994; Dolar et al., 2002; Marsh, Lloze, Heinsohn, & Kasuya, 1989; Minton et al., 2011; Mörzer Bruyns, 1966; Smith & Hobbs, 2002; Smith et al., 2006; Stacey, 1996; Sutaria, 2009). The Trat data may not have been collected far enough from river mouths to be a significant variable in our model. Thus, we may not have recorded a sufficient range of distance to river mouth to properly test the importance of this variable. Trat may represent the ideal habitat for this species, with its access to nutrient‐rich river effluence, given its importance to other small, coastal cetaceans (Hobbie, 2000; McClusky & Elliott, 2006; Rossi‐Santos, Wedekin, & Sousa‐Lima, 2006). Anecdotal evidence suggests that Irrawaddy dolphins were present in Chanthaburi in the past (C. Junchompoo pers. comm.). If we accept these reports, then an alternative explanation for their absence is overfishing in the Gulf of Thailand, for which there is ample evidence (Ahmed, Boonchuwongse, & Dechboon, 2007; Christenson, 1998; Pauly & Chuenpagdee, 2003; Suvapepun, 1991). In addition, Chanthaburi Province and nearby Rayong Province are hubs of industry in the Gulf, part of Thailand’s Eastern Seaboard Development Program (Thailand Government Public Relations Department, 2016). Fishing pressure and industrial development have well‐documented negative effects on marine populations through pollution, defaunation, and other means (e.g., Gimpel, 1976; Goudie, 2013; Constant, Nourry, & Seegmuller, 2014; D’Souza & Peretiatko, 2002; Kalavrouziotis, 2016; Maojun, Jie, Xuechun, & Jin, 2011; McCauley et al., 2015; Nriagu, 1996; Solomon & Palanisami, 2016; Stocker, 2016; Wang, Xu, Sun, Liu, & Li, 2013). Either influence category presented (environmental differences, human encroachment) could explain their absence, but the two need not be mutually exclusive.

**Table 5:** Temperature and depth records from all study areas within and outside of the optimal ranges identified by the model

Data	Variable	Entries in range	Entries out of range
Islands	Temperature	0	137
	Depth	23	114
Chanthaburi	Temperature	8	30
	Depth	10	26
All Trat	Temperature	230	864
	Depth	625	469
Trat 2014	Temperature	146	40
	Depth	103	82

**Table 6:** Average, minimum, and maximum values of distance to river mouth in each area

Variable	Area	Average	Minimum	Maximum
Distance to river mouth (km)	Trat	7.58	0.65	15.34
	Islands	26.62	4.18	47.7
	Chanthaburi	10.58	2.79	17.21

### 4.4 Model caveats and next steps

Using models to guide management decisions requires some assessment of uncertainties. Of concern are low AUC and ρ^2^ values (Table S2), even after repeated model improvements. Such universally low values in multiple iterative tests could be due to missing covariates. Other variables such as prey distributions have been utilized in other SDMs; however, collecting prey data at appropriate scales for modeling efforts is quite difficult (Becker et al., 2016; Hazen & Johnston, 2010; Hyrenbach et al., 2000; Torres et al., 2008). Collection of additional covariates such as finer‐resolution bathymetry data could perhaps allow a more detailed model, but bathymetric data at such a fine scale are less commonly available. Data on human use, specifically fishing effort, could provide an additional limiting factor for dolphin distribution given the observed absences near the islands and Chanthaburi. Such data for our field sites have been collected and analyzed (Jackson‐Ricketts, 2017). However, given the paucity of data for this species, this is the first SDM that can be used to inform conservation options for this population of Irrawaddy dolphins.

These results can also be extended to other Irrawaddy dolphin populations where no habitat data exist. Such locations are spread across South and Southeast Asia, in Brunei, Cambodia, India, Indonesia, Malaysia, the Philippines, Singapore, and Thailand (Adulyanukosol, 1999; Anderson & Kinze, 1999; Chasen, 1940; Dolar et al., 1997; Hines, Junchompoo, Ilangakoon, Ponnampalam, & Jackson‐Ricketts, 2014; Jaaman, 2000; Mörzer Bruyns, 1966; Perrin et al., 2005; Ponnampalam, 2012; Pilleri & Gihr, 1974; Ratnam, 1982). Our results can be used to optimize exploratory surveys in poorly understood areas to determine if the species is present, with the understanding that extrapolation to unstudied areas is only as accurate as the relationships between species and habitat variables are similar (Manocci et al., 2016; Wenger & Olden, 2012).

Our understanding of this species would greatly benefit from development of additional species distribution models for the bays, deltas, and coastal areas where this species is known to occur. Additional models can inform key predictor variables and provide insight on variability in habitat preferences across the species’ range. In addition, models can provide aid in the form of preliminary information on sympatric species. Indo‐Pacific humpback dolphins (*Sousa chinensis*) and Indo‐Pacific finless porpoises (*Neophocaena phocaenoides*) share habitat with Irrawaddy dolphins along the Trat Province coast (Hines et al., 2015). Any form of spatial management undertaken in this area must account for these species, ensuring that spatial management for Irrawaddy dolphins considers the needs of these other cetaceans.

## AUTHORS CONTRIBUTIONS

J. Jackson‐Ricketts, C. Junchompoo, E. Hines, L. Ponnampalam, A. Ilangakoon, and S. Monanunsap collected the data used in this manuscript. J. Jackson‐Ricketts, E. Hines, and E. Hazen chose and performed the statistical analyses. J. Jackson‐Ricketts and E. Hines obtained funding. J. Jackson‐Ricketts wrote the manuscript with input from C. Junchompoo, E. Hines, E. Hazen, L. Ponnampalam, and A. Ilangakoon. C. Junchompoo, E. Hines, and S. Monanunsap acted as principal investigators for a larger project of which this manuscript is one part.

## Data Availability

Data underlying this article will be available on Dryad, with https://doi.org/10.5061/dryad.r2280gb94.

## References

[ece36023-bib-0001] Adulyanukosol, K. (1999). Dugong, dolphin, and whale in Thai waters. Proceedings of the 1st Korea‐Thailand Joint Workshop on Comparison of Coastal Environment: Korea‐Thailand. Sept. 9–10 (1999). Hoam Convention Center. Seoul, Korea: Seoul National University.

[ece36023-bib-0002] Aggarwal, C. C. (2013). Outlier analysis. New York, NY: Springer 10.1007/978-1-4614-6396-2

[ece36023-bib-0003] Ahmed, M. , Boonchuwongse, P. , Dechboon, W. , & Squires, D. (2007). Overfishing in the Gulf of Thailand: Policy challenges and bioeconomic analysis. Environment and Development Economics, 12, 145–172. 10.1017/S1355770X06003433

[ece36023-bib-0004] Alfons, A. (2012). cvTools: Cross‐validation tools for regression models. R Package Version 0.3.2.

[ece36023-bib-0005] Anderson, M. , & Kinze, C. C. (1999). Annotated checklist and identification key to the whales, dolphins, and porpoises (Order Cetacea) of Thailand and adjacent waters. Natural History Bulletin of the Siam Society, 47, 27–62.

[ece36023-bib-0006] Araújo, M. B. , Pearson, R. G. , Thuiller, W. , & Erhard, M. (2005). Validation of species‐climate impact models under climate change. Global Change Biology, 11, 1–10. 10.1111/j.1365-2486.2005.01000.x

[ece36023-bib-0007] Bailey, H. , & Thompson, P. M. (2009). Using marine mammal habitat modelling to identify priority conservation zones within a marine protected area. Marine Ecology Progress Series, 378, 279–287. 10.3354/meps07887

[ece36023-bib-0008] Baird, I. G. , & Beasley, I. L. (2005). Irrawaddy dolphin *Orcaella brevirostris* in the Cambodian Mekong River: An initial survey. Oryx, 39(3), 301–310.

[ece36023-bib-0009] Baird, I. G. , & Mounsouphom, B. (1994). Irrawaddy dolphins (*Orcaella brevirostris*) in Southern Lao PDR and Northwestern Cambodia. Natural History Bulletin of SIAM Soc., 42, 159–175.

[ece36023-bib-0010] Barry, S. , & Elith, J. (2006). Error and uncertainty in habitat models. Journal of Applied Ecology, 43(3), 413–423. 10.1111/j.1365-2664.2006.01136.x

[ece36023-bib-0011] Batisse, M. (1982). The biosphere reserve: A tool for environmental conservation and management. Environmental Conservation, 9(2), 101–112. 10.1017/S0376892900019937

[ece36023-bib-0012] Bearzi, G. , Fortuna, C. M. , & Reeves, R. R. (2008). Ecology and conservation of common bottlenose dolphins *Tursiops truncates* in the Mediterranean Sea. Mammal Review, 39(2), 92–123.

[ece36023-bib-0013] Bearzi, G. , Reeves, R. R. , Notarbartolo‐di‐Sciara, G. , Politi, E. , Cañadas, A. , Frantzis, A. , & Mussi, B. (2003). Ecology, status and conservation of short‐beaked common dolphins *Delphinus delphis* in the Mediterranean Sea. Mammal Review, 33, 224–252. 10.1046/j.1365-2907.2003.00032.x

[ece36023-bib-0015] Beasley, I. , & Davidson, P. J. A. (2007). Conservation Status of Marine Mammals in Cambodian Waters, Including Seven New Cetacean Records of Occurrence. Aquatic Mammals, 33(3), 368–379.

[ece36023-bib-0016] Beasley, I. , Somany, P. , Gilbert, M. , Phothitay, C. , Saksang, Y. , San, L.K. , & Sokha, K. (2007).Review of the status and conservation of Irrawaddy dolphins *Orcaella brevirostris* in the Mekong River of Cambodia, Lao PDR and Vietnam In: Smith B. D., Shore R. G., & Lopez A. (eds) Status and conservation of freshwater populations of Irrawaddy Dolphins. Wildlife Conservation Society Working Paper No. 31. May 2007. 119 pp.

[ece36023-bib-0017] Becker, E. A. , Forney, K. A. , Fiedler, P. C. , Barlow, J. , Chivers, S. J. , Edwards, C. A. , … Redfern, J. V. (2016). Moving towards dynamic ocean management: How well do modeled ocean products predict species distributions? Remote Sensing, 8(2), 149 10.3390/rs8020149

[ece36023-bib-0018] Bräger, S. , Harraway, J. A. , & Manly, B. F. J. (2003). Habitat selection in a coastal dolphin species (*Cephalorhynchus hectori*). Marine Biology, 143, 233–244. 10.1007/s00227-003-1068-x

[ece36023-bib-0019] Brotons, L. , Thuiller, W. , Araújo, M. B. , & Hirzel, A. H. (2004). Presence‐absence versus presence‐only modelling methods for predicting bird habitat suitability. Ecography, 27, 437–448. 10.1111/j.0906-7590.2004.03764.x

[ece36023-bib-0020] Cañadas, A. , Sagarminaga, R. , De Stephanis, R. , Urquiola, E. , & Hammond, P. S. (2005). Habitat preference modelling as a conservation tool: Proposals for marine protected areas for cetaceans in southern Spanish waters. Aquatic Conservation: Marine and Freshwater Ecosystems, 15, 495–521. 10.1002/aqc.689

[ece36023-bib-0023] Chasen, F. N. (1940). A handlist of Malaysian mammals. Bulletin of the Raffles Museum, 15, 108–110.

[ece36023-bib-0024] Christenson, V. (1998). Fishery‐induced changes in a marine ecosystem: Insight from models of the Gulf of Thailand. Journal of Fish Biology, 53, 128–142. 10.1111/j.1095-8649.1998.tb01023.x

[ece36023-bib-0025] Constant, K. , Nourry, C. , & Seegmuller, T. (2014). Population growth in polluting industrialization. Resource and Energy Economics, 36, 229–247. 10.1016/j.reseneeco.2013.05.004

[ece36023-bib-0028] D’Souza, C. , & Peretiatko, R. (2002). The nexus between industrialization and environment: A case study of Indian enterprises. Environmental Management and Health, 13(1), 80–97. 10.1108/09566160210417859

[ece36023-bib-0029] Day, J. , Dudley, N. , Hockings, M. , Holmes, G. , Laffoley, D. , Stolton, S. , & Wells, S. (2012). Guidelines for applying the IUCN protected area management categories to marine protected areas (p. 36). Gland, Switzerland: IUCN.

[ece36023-bib-0030] R Development Core Team . (2009–2013). R: A language and environment for statistical computing. Vienna, Austria: R Foundation for Statistical Computing http://www.R-project.org/.

[ece36023-bib-0031] Dolar, M. L. L. , Perrin, W. F. , Gaudiano, J. P. , Yaptinchay, A. A. S. P. , & Tan, J. M. L. (2002). Preliminary report on a small estuarine population of Irrawaddy dolphins *Orcaella brevirostris* in the Philippines. The Raffles Bulletin of Zoology, (suppl 10), 155–160.

[ece36023-bib-0032] Dolar, M. L. L. , Perrin, W. F. , Yaptinchay, A. A. S. P. , Jaaman, S. A. B. H. J. , Santos, M. D. , Alava, M. N. , & Suliansa, M. S. B. (1997). Preliminary investigations of marine mammal distribution, abundance, and interactions with humans in the southern Sulu Sea. Asian Marine Biology, 14, 61–81.

[ece36023-bib-0033] Dray, S. , Dufour, A.‐B. , & Thioulouse, J. (2016). ade4: Analysis of ecological data: Exploratory and euclidean methods in environmental sciences. R package version 1.7‐4.

[ece36023-bib-0034] Elith, J. , H. Graham, C. , P. Anderson, R. , Dudík, M. , Ferrier, S. , Guisan, A. , … E. Zimmermann, N. (2006). Novel methods improve prediction of species’ distributions from occurrence data. Ecography, 29, 129–151. 10.1111/j.2006.0906-7590.04596.x

[ece36023-bib-0035] Elith, J. , & Leathwick, J. R. (2009). Species distribution models: Ecological explanation and prediction across space and time. Annual Review of Ecology, Evolution, and Systematics, 40, 677–697. 10.1146/annurev.ecolsys.110308.120159

[ece36023-bib-0036] Embling, C. B. , Gillibrand, P. A. , Gordon, J. , Shrimpton, J. , Stevick, P. T. , & Hammond, P. S. (2009). Using habitat models to identify suitable sites for marine protected areas for harbor porpoises (*Phocoena phocoena*). Biological Conservation. 143, 267-279. 10.1016/j.biocon.2009.09.005

[ece36023-bib-0037] ESRI. (2014). ArcGIS desktop: Release 10.3. Redlands, CA: Environmental Systems Research Institute.

[ece36023-bib-0038] Franklin, J. (2009). Mapping species distributions. New York, NY: Cambridge University Press. ISBN 978‐0‐521‐70002‐3.

[ece36023-bib-0039] Gimpel, J. (1976). Medieval machine: The industrial revolution of the middle ages. New York, NY: Holt, Rinehart and Winston. ISBN 0140045147.

[ece36023-bib-0040] Goetz, K. T. , Montgomery, R. A. , Ver Hoef, J. M. , Hobbs, R. C. , & Johnson, D. S. (2012). Identifying essential summer habitat of the endangered beluga whale *Delphinapterus leucas* in Cook Inlet, Alaska. Endangered Species Research, 16, 135–147. 10.3354/esr00394

[ece36023-bib-0042] Gowan, T. A. , & Ortega‐Ortiz, J. G. (2014). Wintering habitat model for the North Atlantic right whale (*Eubalaena glacialis*) in the Southeastern United States. PLoS ONE, 9(4), e95126 10.1371/journal.pone.0095126 24740091 PMC3989274

[ece36023-bib-0043] Goudie, A. S. (2013). The Human Impact on the Natural Environment: Past, Present, and Future, 7th ed. West Sussex, UK: John Wiley & Sons Ltd. ISBN 978-1-118-57657-1.

[ece36023-bib-0044] Guisan, A. , & Thuiller, W. (2005). Predicting species distribution: Offering more than simple habitat models. Ecology Letters, 8, 993–1009. 10.1111/j.1461-0248.2005.00792.x 34517687

[ece36023-bib-0046] Habtemariam, B. T. , & Fang, Q. (2016). Zoning for a multiple‐use marine protected area using spatial multi‐criteria analysis: The case of the Sheik Seid Marine National Park in Eritrea. Marine Policy, 63, 135–143. 10.1016/j.marpol.2015.10.011

[ece36023-bib-0050] Hazen, E. L. , & Johnston, D. J. (2010). Latitudinal complexity in the deep scattering layers and top predator distribution in the Central Equatorial Pacific. Fisheries Oceanography, 19, 427–433.

[ece36023-bib-0052] Hines, E. , Junchompoo, C. , Ilangakoon, A. , Ponnampalam, L. , & Jackson‐Ricketts, J. (2014). Coastal cetaceans in Trat Province, Eastern Thailand (2012–2014). Australia: Final Report to Ocean Park Conservation Foundation http://rtc.sfsu.edu/research/in_hines.html

[ece36023-bib-0054] Hines, E. , Strindberg, S. , Junchompoo, C. , Ponnampalam, L. S. , Ilangakoon, A. D. , Jackson‐Ricketts, J. , & Mananunsap, S. (2015). Line transect estimates of Irrawaddy dolphin abundance along the eastern Gulf Coast of Thailand. Frontiers in Marine Science, 2, 63 10.3389/fmars.2015.00063

[ece36023-bib-0055] Hobbie, J. E. (2000). Estuarine Science: The key to progress in coastal ecological research In Hobbie J. E. (Ed.), Estuarine Science: A synthetic approach to research and practice (pp. 1–11). Washington, DC: Island Press.

[ece36023-bib-0056] Hodge, V. , & Austin, J. (2004). A survey of outlier detection methodologies. Artificial Intelligence Review, 22(2), 85–126. 10.1023/B:AIRE.0000045502.10941.a9

[ece36023-bib-0057] Hooker, S. K. , Whitehead, H. , & Gowans, S. (1999). Marine protected area design and the spatial and temporal distribution of cetaceans in a submarine canyon. Conservation Biology, 13(3), 592–602. 10.1046/j.1523-1739.1999.98099.x

[ece36023-bib-0058] Hothorn, T. , Zeileis, A. , Farebrother, R. W. , Cummins, C. , Millo, G. , & Mitchell, D. (2015). lmtest: Testing linear regression models. R package version 0.9‐34.

[ece36023-bib-0060] Hu, M.‐C. , Pavlicova, M. , & Nunes, E. V. (2011). Zero‐inflated and hurdle models of count data with extra zeros: Examples from an HIV‐risk reduction intervention trial. The American Journal of Drug and Alcohol Abuse, 37(5), 367–375. 10.3109/00952990.2011.597280 21854279 PMC3238139

[ece36023-bib-0062] Hyrenbach, K. D. , Forney, K. A. , & Dayton, P. K. (2000). Marine protected areas and ocean basin management. Aquatic Conservation: Marine and Freshwater Ecosystems, 10, 437–458. 10.1002/1099-0755(200011/12)10:6<437:AID-AQC425>3.0.CO;2-Q

[ece36023-bib-0063] IUCN Standards and Petitions Subcommittee . (2013). Guidelines for using the IUCN red list categories and criteria. version 10.1. Prepared by the Standards and Petitions Subcommittee. Downloadable from http://www.iucnredlist.org/documents/RedListGuidelines.pdf

[ece36023-bib-0064] Jaaman, S. A. (2000). Report on status and conservation of dugongs and inshore cetaceans in Sabah. Working paper, Sulu‐Sulawesi Marine Ecoregion Project Meeting, 26 September 2000, Universiti Malaysia Sabah, Kota Kinabalu.

[ece36023-bib-0066] Jackman, S. , Tahk, A. , Zeileis, A. , Maimone, C. , & Fearon, J. (2015). pscl: Political science computational laboratory, Stanford University. R Package Version 1.4.9.

[ece36023-bib-0067] Jackson‐Ricketts, J. (2017). Diet, life history, habitat, and conservation of Irrawaddy dolphins, *Orcaella brevirostris,* in the Gulf of Thailand (PhD thesis). Santa Cruz, USA: University of California.

[ece36023-bib-0068] Johnson, J. B. , & Omland, K. S. (2004). Model selection in ecology and evolution. TRENDS in Ecology and Evolution, 19(2), 101–108. 10.1016/j.tree.2003.10.013 16701236

[ece36023-bib-0070] Kadane, J. B. , & Lazar, N. A. (2003). Methods and criteria for model selection. Journal of the American Statistical Association, 99(465), 279–290. 10.1198/016214504000000269

[ece36023-bib-0071] Kalavrouziotis, I. K. (2016). Bioaccumulation due to industrialization. Journal of Environmental & Analytical Toxicology, 6(3), 106 10.4172/2161-0525.1000e106

[ece36023-bib-0072] Karczmarski, L. (2000). Conservation and management of humpback dolphins: The South African perspective. Oryx, 34(3), 207–216.

[ece36023-bib-0073] Kelleher, G. (1999). Guidelines for marine protected areas. Best practice protected area guidelines series no. 3. Gland: World Commission on Protected Areas of IUCN.

[ece36023-bib-0075] Kohavi, R. (1995). A study of cross‐validation and bootstrap for accuracy estimation and model selection. International Joint Conference on Artificial Intelligence. 2, 1137-1143.

[ece36023-bib-0076] Kreb, D. , & Budiono . (2005). Cetacean diversity and habitat preferences in tropical waters of East Kalimantan. Indonesia. the Raffles Bulletin of Zoology, 53(1), 149–155.

[ece36023-bib-0077] Kreb, D. , & Rahadi, K. D. (2004). Living under an aquatic freeway: Effects of boats on Irrawaddy dolphins (*Orcaella brevirostris*) in a coastal and riverine environment in Indonesia. Aquatic Mammals, 30(3), 363–375.

[ece36023-bib-0078] Lausche, B. (2011). Guidelines for protected area legislation. Gland, Switzerland: IUCN. xxvi+370.

[ece36023-bib-0081] Manocci, L. , Roberts, J. J. , Miller, D. L. , & Halpin, P. N. (2016). Extrapolating cetacean densities to quantitatively assess human impacts on populations in the high seas. Conservation Biology. 31, 601-614. 10.1111/cobi.12856 PMC5435923 27775847

[ece36023-bib-0082] Maojun, W. , Jie, X. , Xuechun, Y. , & Jin, W. (2011). The research on the relationship between industrial development and environmental pollutant emission. Energy Procedia, 5, 555–561. 10.1016/j.egypro.2011.03.097

[ece36023-bib-0083] Marsh, H. , Lloze, R. , Heinsohn, G. E. , & Kasuya, T. (1989).Irrawaddy Dolphin *Orcaella brevirostris* (Gray, 1866) In Ridgeway S. H. & Harrison R. J. (Eds.), Handbook of marine mammals (Vol. 4). London: Academic Press.

[ece36023-bib-0084] McCauley, D. J. , Pinsky, M. L. , Palumbi, S. R. , Estes, J. A. , Joyce, F. H. , & Warner, R. R. (2015). Marine defaunation: Animal loss in the global ocean. Science, 347(6219), 1255641 10.1126/science.1255641 25593191

[ece36023-bib-0085] McClusky, D. S. , & Elliott, M. E. (2006). Estuarine ecosystem: Ecology, threats and management (3rd ed). New York, NY: Oxford University Press.

[ece36023-bib-0086] McFadden, D. (1978). Chapter 13: Quantitative methods for analysing travel behaviour of individuals: Some recent developments In: Hensher D. A. & Stopher P. R. (Eds.), Behavioural travel modelling. London, UK: Croom Helm.

[ece36023-bib-0087] Minton, G. , Peter, C. , Poh, A. N. Z. , Ngeian, J. , Braulik, G. , Hammond, P. S. , & Tuen, A. A. (2013). Population estimates and distribution patterns of Irrawaddy dolphin (*Orcaella brevirostris*) and Indo‐Pacific finless porpoises (*Neophocaena phocaenoides*) in the Kuching Bay. Sarawak. The Raffles Bulletin of Zoology, 61(2), 877–888.

[ece36023-bib-0088] Minton, G. , Peter, C. , & Tuen, A. A. (2011). Distribution of small cetaceans in the nearshore waters of Sarawak, East Malaysia. The Raffles Bulletin of Zoology, 59(1), 91–100.

[ece36023-bib-0089] Minton, G. , Smith, B. D. , Braulik, G. T. , Kreb, D. , Sutaria, D. , & Reeves, R. (2017). Orcaella brevirostris. The IUCN Red List of Threatened Species 2017 Retrieved Jan 30, 2018, from http://www.iucnredlist.org

[ece36023-bib-0091] Morin, X. , & Thuiller, W. (2009). Comparing niche‐ and process‐based models to reduce prediction uncertainty in species range shifts under climate change. Ecology, 90(5), 1301–1313. 10.1890/08-0134.1 19537550

[ece36023-bib-0092] Mörzer Bruyns, W. F. J. (1966). Some notes on the Irrawaddy dolphin, *Orcaella brevirostris* (Owen 1866). Zeitschrift Fur Säugetierkunde, 31, 367–372.

[ece36023-bib-0095] Naimi, B . (2015). usdm: Uncertainty analysis for species distribution models. R package version 1.1‐15.

[ece36023-bib-0096] Nriagu, J. O. (1996). A history of global metal pollution. Science, 272(5259), 223 10.1126/science.272.5259.223

[ece36023-bib-0099] Paradis, E. , Bomberg, S. , Bolker, B. , Claude, J. , Cuong, H. S. , Desper, R. , … de Vienne, D. (2015). ape: Analyses of phylogenetics and evolution. R Package Version 3.4.

[ece36023-bib-0101] Parra, G. J. , Corkeron, P. J. , & Marsh, H. (2006). Population sizes, site fidelity and residence patterns of Australian snubfin and Indo‐Pacific humpback dolphins: Implications for conservation. Biological Conservation, 129, 167–180. 10.1016/j.biocon.2005.10.031

[ece36023-bib-0102] Pattnaik, A. K. , Sutaria, D. , Khan, M. , & Behera, B. P. (2007).Review of the status and conservation of Irrawaddy dolphins Orcaella brevirostris in Chilika Lagoon of India In Smith B. D., Shore R. G., & Lopez A. (Eds.), Status and conservation of freshwater populations of Irrawaddy dolphins. Wildlife Conservation Society Working Paper No. 31. May 2007. 119 pp.

[ece36023-bib-0103] Pauly, D. , & Chuenpagdee, R. (2003). Development of fisheries in the Gulf of Thailand large marine ecosystem: Analysis of an unplanned experiment In Hempeland G. & Sherman K. (Eds.), Large marine ecosystems of the world 12: Change and sustainability. Amsterdam: Elsevier Science.

[ece36023-bib-0104] Perrin, W. F. , Reeves, R. R. , Dolar, M. L. L. , Jefferson, T. A. , Marsh, H. , Wang, J. Y. , & Estacion, J. (2005). Report of the second workshop on the biology and conservation of small cetaceans and Dugongs of South‐East Asia. CMS technical series (p. 162). Dumaguete City, Philippines: Silliman University.

[ece36023-bib-0106] Peter, C. , Poh, A. N. Z. , Ngeian, J. , Tuen, A. A. , & Minton, G. (2016). Identifying habitat characteristics and critical areas for Irrawaddy dolphin, *Orcaella brevirostris*: Implications for conservationIn Dasi I. & Tuen A. A. (Eds.), Naturalists, explorers and field scientists in South‐East Asia and Australasia. Topics in Biodiversity and Conservation 15. 10.1007/978-3-319-26161-4_15.

[ece36023-bib-0107] Pilleri, G. , & Gihr, M. (1974). Contribution to the knowledge of the cetaceans of southwest and monsoon Asia (Persian Gulf, Indus delta, Malabar, Andaman Sea and Gulf of Siam) In Pilleri G. (Ed.), Investigations on Cetacea (Vol. V, pp. 95–149). Berne: G. Pilleri.

[ece36023-bib-0108] Ponnampalam, L. S. (2012). Opportunistic observations of the distribution of cetaceans in Malaysian South China Sea, Sulu and Sulawesi Seas and an updated checklist of marine mammals in Malaysia. The Raffles Bulletin of Zoology, 60(1), 221–231.

[ece36023-bib-0109] Ponnampalam, L. S. (2013). The dolphins of the Matang mangroves In Ariffin R. & Mustafa N., NMS (Eds.), A working plan for the Matang Mangroves Forest Reserve, Perak (6th revision, pp. 110–112). Malaysia: Perak State Forestry Department.

[ece36023-bib-0110] Ponnampalam, L. S. , Kuit, S. H. , & Chong, V. C. (2014). Ecology and conservation of coastal cetaceans in the Matang Mangrove Forest Reserve and adjacent coastal waters, Perak, Peninsular Malaysia, with references to Indo‐Pacific humpback dolphins (*Sousa chinensis*) and Irrawaddy dolphins (*Orcaella brevirostris*). Hong Kong: Final report to Ocean Park Conservation Foundation.

[ece36023-bib-0111] Ratnam, L. C. (1982). The Irrawaddy river dolphin in the Bernam River. The Journal of Wildlife and Parks, 1, 17.

[ece36023-bib-0112] Redfern, J. V. , Ferguson, M. C. , Becker, E. A. , Hyrenbach, K. D. , Good, C. , Barlow, J. , … Werner, F. (2006). Techniques for cetacean‐habitat modelling. Marine Ecology Progress Series, 310, 271–295.

[ece36023-bib-0113] Reeves, R. R. , Jefferson, T. A. , Karczmarski, L. , Laidre, K. , O’Corry‐Crowe, G. , Rojas‐Bracho, L. , … Zhou, K. (2008). Orcaella brevirostris In: IUCN 2011. IUCN Red List of Threatened Species. Version 2011.2. http://www.iucnredlist.org

[ece36023-bib-0114] Refaeilzadeh, P. , Tang, L. , & Liu, H. (2009).Cross validation In Liu L. & Ozsu M. T. (Eds.), Encyclopedia of database systems (pp. 532–538). New York, NY: Springer, US.

[ece36023-bib-0116] Rossi‐Santos, M. , Wedekin, L. L. , & Sousa‐Lima, R. S. (2006). Distribution and habitat use of small cetaceans off Abrolhos Bank, Eastern Brazil. Latin American Journal of Aquatic Mammals, 5(1), 23–28. 10.5597/lajam00088

[ece36023-bib-0117] Sahu, H. K. , Kar, S. K. , & Pattnaik, S. K. (1998). Study on some aspects of Irrawaddy River Dolphin *Orcaella brevirostris* Gray in Chilika Lake. Orissa. The Indian Forester, 124(10), 803–809.

[ece36023-bib-0118] Sing, T. , Sander, O. , Beerenwinkel, N. , & Lengauer, T. (2015). ROCR: Visualizing the performance of scoring classifiers. R package version 1.0‐7.

[ece36023-bib-0119] Smith, B. D. , Ahmed, B. , Mansur, R. , Tun, M. T. , & Tun, T. (2005). New information on the status of finless porpoises Neophocaena phocaenoides and Irrawaddy dolphins *Orcaella brevirostris* in Bangladesh and Myanmar. WCS Report SC/57/SM4, 19 pages.

[ece36023-bib-0120] Smith, B. D. , Beasley, I. , Buccat, M. , Calderon, V. , Evina, R. , de Valle, J. L. , … Visitacion, Z. (2004). Status, ecology and conservation of Irrawaddy dolphins (*Orcaella brevirostris*) in Malampaya Sound, Philippines. Journal of Cetacean Research and Management, 6(1), 41–52.

[ece36023-bib-0121] Smith, B. D. , Beasley, I. , & Kreb, D. (2003). Marked declines in populations of Irrawaddy dolphins. Oryx, 37(4), Chapter 8, 127–131.

[ece36023-bib-0122] Smith, B. D. , Braulik, G. , Strindberg, S. , Ahmed, B. , & Mansur, R. (2006). Abundance of Irrawaddy dolphins (*Orcaella brevirostris*) and Ganges River dolphins (*Platanista gangetica gangetica*) estimated using concurrent counts made by independence teams in waterways of the Sundarbans mangrove forest in Bangladesh. Marine Mammal Science, 22(3), 527–547.

[ece36023-bib-0123] Smith, B. D. , & Hobbs, L. (2002). Status of Irrawaddy dolphins *Orcaella brevirostris* in the upper reaches of the Ayeyarwady River, Myanmar. Raffles Bulletin of Zoology Supplement, 10, 67–73.

[ece36023-bib-0124] Smith, B. D. , & Jefferson, T. A. (2002). Status and conservation of facultative freshwater cetaceans in Asia. Raffles Bulletin of Zoology, Supplement 10. 173–187.

[ece36023-bib-0125] Smith, B. D. , Shore, R. G. , & Lopez, A. (Eds.). (2007). Status and conservation of freshwater populations of Irrawaddy dolphins. Wildlife Conservation Society Working Paper No. 31. May 2007. 119 pp.

[ece36023-bib-0127] Solomon, O. O. , & Palanisami, T. (2016). Microplastics in the marine environment: current status, assessment methodologies, impacts and solutions. Journal of Pollution Effects & Control, 4(2), 161.

[ece36023-bib-0128] St. Martin, K. , & Hall‐Arber, M. (2008). The missing layer: Geo‐technologies, communities, and implications for marine spatial planning. Marine Policy, 32, 779–786. 10.1016/j.marpol.2008.03.015

[ece36023-bib-0129] Stacey, P. J. (1996). Natural History and Conservation of Irrawaddy dolphins, *Orcaella brevirostris*, with special reference to the Mekong River, Lao P.D.R (M.Sc thesis). Victoria, British Columbia: Department of Geography, University of Victoria.

[ece36023-bib-0130] Stacey, P. J. , & Hvengaard, G. T. (2002). Habitat use and behaviour of Irrawaddy dolphins (*Orcaella brevirostris*) in the Mekong River of Laos. Aquatic Mammals, 28(1), 1–13.

[ece36023-bib-0131] Stocker, M. (2016). Potential noise impacts of current and advancing marine technologies in the industrialization of the ocean. 171st Meeting of the Acoustical Society of America, Salt Lake City, Utah, 23–27 May 2016, Proceedings of Meetings on Acoustics, 26, 005001.

[ece36023-bib-0133] Sutaria, D. (2009). Species conservation in a complex socio‐ecological system: Irrawaddy dolphins, *Orcaella brevirostris* in Chilika Lagoon, India (PhD thesis). Townsville, Australia: James Cook University.

[ece36023-bib-0134] Suvapepun, S. (1991). Long terms ecological changes in the Gulf of Thailand. Marine Pollution Bulletin, 23, 213–217.

[ece36023-bib-0135] Teh, L. S. L. , Teh, L. C. L. , Hines, E. , Junchompoo, C. , & Lewison, R. L. (2015). Contextualizing the coupled socio‐ecological conditions of marine megafauna bycatch. Ocean & Coastal Management, 116, 449–465.

[ece36023-bib-0136] Thailand Government Public Relations Department . (2016, 20 June). Thailand’s Eastern Seaboard Development. Office of the National Economic and Social Development Board Retrieved from http://www.nesdb.go.th/ewt_dl_link.php?nxml:id=6473

[ece36023-bib-0137] Torres, L. G. , Read, A. J. , & Halpin, P. (2008). Fine‐scale habitat modeling of a top marine predator: Do prey data improve predictive capacity? Ecological Applications, 18(7), 1702–1717. 10.1890/07-1455.1 18839765

[ece36023-bib-0138] Ver Hoef, J. M. , & Jansen, J. K. (2007). Space‐time zero‐inflated count models of Harbor seals. Environmetrics, 18, 697–712. 10.1002/env.873

[ece36023-bib-0139] Wang, S.‐L. , Xu, X.‐R. , Sun, Y.‐X. , Liu, J.‐L. , & Li, H.‐B. (2013). Heavy metal pollution in coastal areas of South China: A review. Marine Pollution Bulletin, 76, 7–15. 10.1016/j.marpolbul.2013.08.025 24084375

[ece36023-bib-0140] Wenger, S. J. , & Olden, J. D. (2012). Assessing transferability of ecological models: An underappreciated aspect of statistical validation. Methods in Ecology and Evolution, 3, 260–267. 10.1111/j.2041-210X.2011.00170.x

[ece36023-bib-0141] Yanuar, A. , Tjiu, A. , Suprapti, D. , Syahirsyah, I. , Saniswan, Y. , & Widjaya, I. (2011). Mission Report: Discovery of Irrawaddy dolphin *Orcaella brevirostris* population and habitat in Kubu Raya waters, West Kalimantan: A preliminary survey of Irrawaddy dolphin in salt and brackish waters. Report to the Regional Office for Marine, Coastal and Resources Management.

[ece36023-bib-0142] Zeileis, A. , Kleiber, C. , & Jackman, S. (2008). Regression models for count data in R. Journal of Statistical Software, 27(8), 1–25.

[ece36023-bib-0143] Zuur, A. F. , Ieno, E. N. , & Smith, G. M. (2007). Analysing ecological data. New York, NY: Springer Science + Business Media. ISBN‐10: 0‐387‐45967‐7.

[ece36023-bib-0144] Zuur, A. F. , Ieno, E. N. , Walker, N. J. , Saveliev, A. A. , & Smith, G. M. (2009). Mixed effects models and extensions in ecology with R. New York, NY: Springer Science + Business Media. ISBN 978‐0‐387‐87457‐9.

