# Supplementary material for: Habitat modeling of Irrawaddy dolphins (Orcaella brevirostris) in the Eastern Gulf of Thailand
Source: Ecol Evol. 2020 Mar 4;10(6):2778–92. doi: 10.1002/ece3.6023 (PMC7083678; doi:10.1002/ece3.6023)
Supplement: Supplementary file 1 [file ECE3-10-2778-s001.docx]

**Habitat Modeling of Irrawaddy Dolphins (*Orcaella brevirostris*) in the Eastern Gulf of Thailand**

**Supplemental Information**

*Introduction*

Species distribution models have been used in the past to determine the habitat of marine mammals. For example, Bräger, Hararaway, & Manly (2003) used a logistic regression model with a binary response to show temperature, turbidity, and depth preferences of Hector’s dolphins (*Cephalorhynchus hectori*) in New Zealand. Goetz, Montgomery, Ver Hoef, Hobbs, & Johnson (2012) used a hurdle model to show that beluga whales (*Delphinapterus leucas*) in Cook Inlet, Alaska, prefer tidal flats and sandy substrate in the summer. Researchers use assessments of model fit, prediction ability, and variance explained to evaluate discrepancies between model results and true phenomena (McFadden 1978, Kohavi 1995, Kadane and Lazar 2003, Johnson and Omland 2004, Redfern et al. 2006, Zuur, Ieno, & Smith 2007, Franklin 2009, Refaeilzadeh, Tang, & Liu 2009).

*Methods*

Hurdle models have been used to model the distribution of marine mammals, including to examine haulout patterns of harbor seals, wintering habitat of North Atlantic right whales, and summer habitat of belugas (Ver Hoef and Jansen 2007, Goetz et al. 2012, Gowan and Ortega-Ortiz 2014).

We explored three distributions - negative binomial, Poisson, and zero-inflated negative binomial – and compared results using the model selection and evaluation criteria detailed within the main body of the paper.

*Results*

Model selection results are presented in Tables S1 and S2.

Full model fitted values (dolphin occurrence likelihood) show the same functional relationships between depth and temperature as individual probability and predicted number (Figure S1a, b).

**Supplemental Figure**


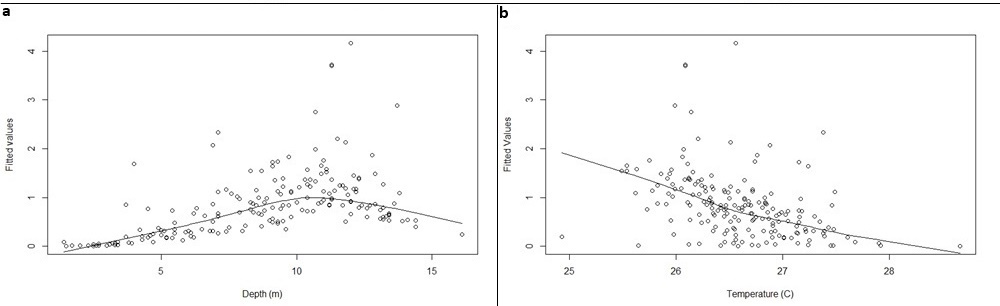


Figure S1: a) the relationship between overall predicted dolphin occurrence likelihood (model fitted values) and depth, and b) the relationship between overall predicted dolphin occurrence likelihood and temperature.

**Supplemental Tables**

Table S1: Model selection results, giving the criteria from the best models of each type from each framework. Single asterisks indicate the best AUC score for that framework. Double asterisks indicate the best overall AUC scores.

| Framework | No. of variables | AIC | CV | LR test | AUC |
| --- | --- | --- | --- | --- | --- |
| 1 |  |  |  |  |  |
| *Negative binomial hurdle* | 5 | 2200.07 | 2.22 | p>>0.05 | *Zero:* 0.644  *Count:* 0.632 |
| *Poisson hurdle* | 5 | 2391.68 | 2.22 | p>0.05 | *Zero:* 0.649*  *Count:* 0.630 |
| *Zero-inflated negative binomial* | 5 | 2207.99 | 2.23 | p>0.05 | *Zero:* 0.645  *Count:* 0.633* |
| 2 |  |  |  |  |  |
| *Negative binomial hurdle* | 4 | 1743.83 | 2.38 | p>>0.05 | *Zero:* 0.562  *Count:* 0.5251 |
| *Poisson hurdle* | 4 | 1932.64 | 2.39 | p>>0.05 | *Zero:* 0.569*  *Count:* 0.522 |
| *Zero-inflated negative binomial* | 4 | 1752.73 | 2.39 | p>>0.05 | *Zero:* 0.561  *Count:* 0.5255* |
| 3 |  |  |  |  |  |
| *Negative binomial hurdle* | 3 | 779.38 | 1.76 | p>0.05 | *Zero:* 0.672  *Count:* 0.635* |
| *Poisson hurdle* | 6 | 805.99 | 1.75 | p>>0.05 | *Zero:* 0.674  *Count:* 0.632 |
| *Zero-inflated negative binomial* | 7 | 786.98 | 1.79 | NA: full model | *Zero:* 0.679**  *Count:* 0.640 |
| 4 |  |  |  |  |  |
| *Negative binomial hurdle* | 4 | 425.35 | 1.82 | p>>0.05 | *Zero:* 0.631  *Count:* 0.681** |
| *Poisson hurdle* | 4 | 438.62 | 1.76 | p>>0.05 | *Zero:* 0.648*  *Count:* 0.665 |
| *Zero-inflated negative binomial* | 1 | 426.87 | 1.73 | p>0.05 | *Zero:* 0.638  *Count:* 0.532 |
| 5 |  |  |  |  |  |
| *Negative binomial hurdle* | 4 | 1902.35 | 2.28 | p>>0.05 | *Zero:* 0.649  *Count:* 0.658 |
| *Poisson hurdle* | 4 | 2093.73 | 2.28 | p>>0.05 | *Zero:* 0.652*  *Count:* 0.658 |
| *Zero-inflated negative binomial* | 4 | 1910.65 | 2.28 | p>>0.05 | *Zero:* 0.650  *Count:* 0.659* |

Table S2: Model selection results of models with quadratic terms. The framework 4 model actually had a higher AUC for the zero component than the framework 3 model. Although the AUC was higher for the count component of the first framework 4, the second-order temperature term was not significant. Asterisks indicate the best AUC scores, while double asterisks indicate the best overall model.

| Framework | Variables in zero model | Variables in count model | AIC | CV | LR test | AUC | ρ^2^ |
| --- | --- | --- | --- | --- | --- | --- | --- |
| 3  quadratic depth | 7 | 6 | 765.54 | 1.73 | p>0.05 | *Zero:* 0.724  *Count:* 0.619 | 0.11 |
| 4  quadratic depth and temp | 5 | 4 | 418.06 | 1.70 | p>0.05 | *Zero:* 0.733  *Count:* 0.759* | 0.13 |
| 4**  quadratic depth | 5 | 4 | 416.48 | 1.71 | p>0.05 | *Zero:* 0.737*  *Count:* 0.746 | 0.13 |

Table S3: Results of the chosen model, showing a significant polynomial relationship between dolphin presence and depth and a significant negative relationship between group size and temperature.

|  | Variable | Estimate | Std. Error | Z value | p value |
| --- | --- | --- | --- | --- | --- |
| Zero component | Intercept | -51.27 | 38.02 | -1.35 | 0.177 |
|  | Salinity | 1.50 | 1.16 | 1.30 | 0.194 |
|  | Turbidity | 1.39 | 0.72 | 1.93 | 0.053 |
|  | Calves | 17.20 | 1476.85 | 0.01 | 0.991 |
|  | 1^st^ order depth | 14.11 | 3.89 | 3.62 | 0.0003*** |
|  | 2^nd^ order depth | -11.82 | 3.78 | -3.12 | 0.002** |
|  | Distance to river mouth | -0.08 | 0.06 | -1.40 | 0.163 |
| Count component | Intercept | 30.90 | 10.03 | 3.08 | 0.002** |
|  | Temperature | -0.95 | 0.33 | -2.86 | 0.004** |
|  | Turbidity | 0.53 | 0.33 | 1.61 | 0.107 |
|  | Chlorophyll *a* | -0.20 | 0.20 | -1.04 | 0.301 |
|  | pH | -0.68 | 0.58 | -1.16 | 0.247 |

Table S4: Existing habitat data, this study included as the last entry. *Studies in which habitat was determined statistically.

| Area | Habitat Type | Depth (m) | Temperature (^o^C) | Turbidity | Salinity (ppt) | pH | Distance to coast (km) | Distance to river mouth (km) | Reference |
| --- | --- | --- | --- | --- | --- | --- | --- | --- | --- |
| Sundarbans, Bangladesh | Delta: outer | Mean: 7.5  Range: 2.7-16 | Mean: 23.7  Range: 21.8-25.4 | Mean: 295 NTU  Range: 8.5-3079 | Mean: 16.1  Range: 7-34 | na | na | na | Smith et al. 2005 |
| Cambodia | Coastal | Mean: 8  Range: 1.6-16.7 | na | na | na | na | na | na | Beasley and Davidson 2007 |
| East Kalimantan, Indonesia | Bay | Mean: 14.3  Range: 2.5-30 | na | na | na | na | na | na | Kreb and Budiono 2005a |
| East Kalimantan, Indonesia | Coastal | Mean: 6.9  Range: 2-23 | na | na | na | na | na | na | Kreb and Budiono 2005a |
| East Kalimantan, Indonesia | Delta | Mean: 5.6  Range: 3-10 | na | na | na | na | na | na | Kreb and Budiono 2005a |
| Mahakam Delta, Indonesia | Delta | Mean: 5.6  Range: 3-10 | na | na | Mean: 12  Range: 4.6-19.3 | na | na | na | Kreb and Budiono 2005a |
| Balikpapan Bay, Indonesia | Bay | Mean: 14.5  Range: 2-30 | na | Mean: 170 cm | na | na | na | na | Kreb and Rahadi 2004 |
| Perak, Malaysia | Coastal and Estuary | Mean: 5.57  Range: 0.9-14.4 | Mean: 29.71  Range: 26.7-31.2 | na | Mean: 29.61  Range: 19.97-34.52 | Mean: 8.16  Range: 6.46-10.6 | na | na | Ponnampalam 2012, 2013, Ponnampalam et al. 2014 |
| Sarawak, Malaysia | Coastal | Mean: 3.3  Range: 2-5.4 | na | na | na | na | Mean: 1.38 | Mean: 3.53 | Minton et al. 2011 |
| Kuching Bay, Malaysia* | Bay | na | na | na | Optimal: 28-30.99 | na | na | ≤6 | Minton et al. 2013, Peter et al. 2016 |
| Mergui Archipelago, Myanmar | Offshore | 18.8 | 30.2 | 4 NTU | 31.8 | na | na | na | Smith et al. 2005 |
| Malampaya Sound, Philippines | Coastal | Mean: 6.5  Range: 1.5-15.1 | Mean: 30.2  Range: 27-32.5 | Mean: 2.2 NTU  Range: 0-9.6 | Mean: 28.3  Range: 14-34 | na | na | na | Dolar et al. 2002, Smith et al. 2004 |
| Gulf of Thailand* | Coastal | Optimal: ~10  Range: 7.5-13.05 | Optimal: 24.93  Range: 24.93-26.99 | na | na | na | na | na | This study |
